# Survey-based Work System Assessment to Facilitate Large-scale Dissemination of Healthcare Quality Improvement Programs

**DOI:** 10.1097/pq9.0000000000000288

**Published:** 2020-04-09

**Authors:** Anping Xie, Danielle W. Koontz, Annie Voskertchian, James C. Fackler, Aaron M. Milstone, Charlotte Z. Woods-Hill

**Affiliations:** From the * Armstrong Institute for Patient Safety and Quality, Johns Hopkins University School of Medicine, Baltimore, Md.; † Department of Anesthesiology and Critical Care Medicine, Johns Hopkins University School of Medicine, Baltimore, Md.; ‡ Department of Pediatrics, Division of Infectious Diseases, Johns Hopkins University School of Medicine, Baltimore, Md.; § Department of Epidemiology, Johns Hopkins Bloomberg School of Public Health, Baltimore, Md.; ¶ Department of Anesthesiology and Critical Care Medicine, The Children’s Hospital of Philadelphia, Philadelphia, Pa.

## Abstract

**Introduction:**

The dissemination of quality improvement (QI) interventions to a broader range of healthcare settings requires a proactive assessment of local work systems and processes. The objective of this study was to examine the feasibility of using a survey-based work system assessment (WSA) tool to facilitate the dissemination of a program for optimizing blood culture (BC) use.

**Methods:**

Informed by findings from an onsite, interview-based WSA at 2 hospitals, a 50-item WSA survey was devised and administrated to 15 hospitals participating in a QI collaborative. WSA survey data were summarized, shared, and discussed with individual hospitals to inform the adaptation and implementation of the BC program. Physician champions leading the local QI team assessed the use of the WSA survey by completing an 8-item survey.

**Results:**

A total of 347 clinicians completed the WSA survey, and physician champions at 12 hospitals evaluated the use of the WSA survey. Both the WSA survey data and the evaluation of the WSA survey showed that the survey-based WSA tool could help participating hospitals understand their current BC ordering practices and identify potential barriers to implementing the program from the perspectives of different clinicians.

**Conclusions:**

We demonstrated how a survey-based tool could be used to facilitate WSA in the dissemination of a program for improving BC use to a multisite collaborative. A survey-based WSA tool can be used to facilitate future large-scale intervention dissemination efforts.

## Abstract 2

Supplemental Digital Content is available in the text.

## INTRODUCTION

Although great efforts have been made to improve healthcare quality and safety,^1–3^ the dissemination of successful quality improvement (QI) interventions to a broad range of settings is neither straightforward nor spontaneous.^4^ Every setting has its unique context, which requires the customization of interventions and implementation strategies. A proactive assessment of local work systems and processes, therefore, is essential.^5^ A previous study showed how a work system assessment (WSA) based on qualitative interviews with local stakeholders could facilitate the dissemination of a QI program for optimizing blood culture (BC) use in pediatric intensive care units (PICUs) at 2 hospitals.^6^ However, given the time and resources required, an interview-based WSA may not be feasible for large-scale dissemination. This article describes a modified approach to WSA (ie, survey-based WSA) and its application to the spread of the QI program for optimizing BC use to a multisite collaborative.

### The *B*lood Cultu*r*e *I*mprovement *G*uidelines and Diagnos*t*ic *St*ewardship for *A*ntibiotic *R*eduction in Critically Ill Children (Bright STAR) Collaborative

In 2014, Johns Hopkins Hospital initiated a QI program to improve BC ordering practices in the PICU. As part of the program, a clinical decision support tool was developed to guide clinicians to consider possible sources of infection, evaluate noninfectious sources of fever, and carefully review risk factors of patients for bloodstream infections. The implementation of the program resulted in a reduction in the total number of BCs collected by 46%.^7^ Two additional hospitals then decided to adopt the program. The research team performed a WSA by interviewing different stakeholders (eg, physicians and nurses) during a 2-day trip to each hospital.^6^ Informed by findings of the WSA, the 2 hospitals adapted the program to their context and achieved a 21%−37% reduction in BC use.^8^

Given the initial success of the program, a multisite collaborative called Bright STAR was created to scale-up the program and assess its broader impact on BC use and patient safety. The Bright STAR collaborative includes 15 hospitals across 14 states whose PICUs vary in size and patient volume. Each participating hospital convened a local QI team, led by 2 physician champions, and involving many other stakeholders. The research team routinely communicated with the local QI teams through various avenues, including an initial individual site call, monthly individual site calls, monthly all-site calls, and individual and group emails. Because of the number and geographic span of the participating hospitals, the research team could not visit each hospital and conduct in-person interviews. With limited capabilities and resources, the local QI teams could also not easily conduct their own WSA. Therefore, the research team adapted the onsite, interview-based WSA to a survey-based WSA. In this article, we examine the feasibility of using the survey-based WSA for large-scale dissemination.

## METHODS

### WSA Survey Design

Based on the interview-based WSA,^6^ the research team with experts in infectious diseases, critical care, QI, and human factors engineering devised a WSA survey (**see Supplemental Digital Content** at http://links.lww.com/PQ9/A177) using the Qualtrics survey software (Qualtrics Labs Inc., Provo, Utah). The survey addressed 3 areas: (1) perceptions of current BC ordering practices; (2) beliefs about good BC ordering practices; and (3) potential barriers to reducing unnecessary BCs. Most questions were assessed using 5-point Likert scales. Additional information about provider type, years of experience, and the associated institution was collected. The survey was pilot tested with PICU clinicians at Johns Hopkins Hospital and the Children’s Hospital of Philadelphia and revised based on the feedback received.

### WSA Survey Administration

We administered the WSA survey to all 15 hospitals after the initial individual site calls. An email with a link to the survey was sent to the physician champions at each hospital, who then shared the link with other PICU clinicians within their institution. The responses from each hospital were continuously monitored. If a hospital had <15 responses or had no responses from specific groups (eg, nurses), the research team would contact the physician champions to send further reminders to their PICU clinicians.

### Analysis of WSA Survey Data

We analyzed survey data using descriptive statistics. For each hospital, the proportion of respondents who reported certain practices were summarized. Questions with 5-point Likert scales were categorized as positive responses by combining “agree” and “strongly agree” or “somewhat likely” and “extremely likely,” and as negative responses by combining “disagree” and “strongly disagree” or “somewhat unlikely” and “extremely unlikely.” Results are presented as medians and interquartile ranges across all hospitals.

### Sharing of WSA Survey Data

The WSA survey results were shared with each hospital during a dedicated individual site call. We created and tailored a template slide presentation to present the unique findings from each hospital, as well as the common findings across hospitals. During the call, the research team reviewed the slide presentation with the local QI team and encouraged them to ask questions and share any insights about the data under review. This call, in turn, promoted a discussion of how to use the WSA survey results to facilitate the implementation of the program. Following the call, the research team sent a copy of the site-specific slide presentation along with a summary of the discussion to the local QI team. Finally, we shared the aggregated WSA survey results reflecting responses across all 15 hospitals during an all-site call.

### Evaluation of the Use of the WSA Survey

After completion of all WSA-focused individual site calls, the physician champions at each hospital provided feedback about the use of the survey-based WSA through a short survey that was completed during one of the monthly conference calls with the research team. The survey included 8 questions with response categories on a 5-point Likert scale (Table 1). Also, qualitative comments on the use of the survey-based WSA were collected.

**Table 1.**
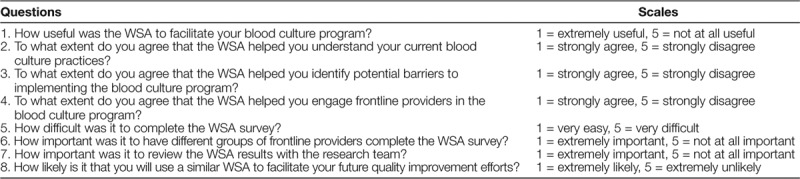
Questions for Evaluating the WSA Survey

## RESULTS

### WSA Survey Data

Table 2 shows the number of respondents of the WSA survey from each hospital. Table 3 summarizes the results of selected WSA survey questions.

**Table 2.**
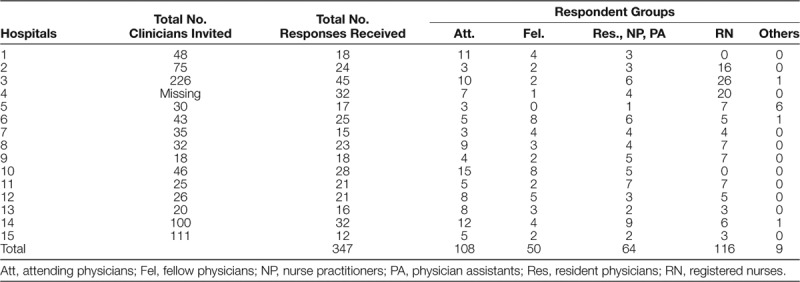
Respondents of the WSA Survey

**Table 3.**
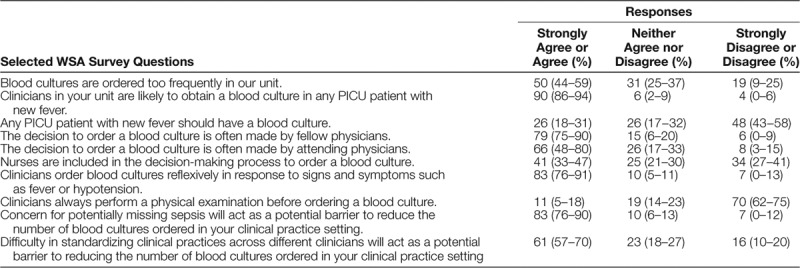
Summary of WSA Survey Data

Overall, half of the respondents from each hospital thought that BCs are ordered too frequently on their units (median proportion of respondents, 50%; interquartile range, 44%–59%). The main reason for BC overuse was collecting BCs for all PICU patients with new fever [90% (86%–94%)]. We observed discrepancies between perceived clinical practices and the theoretical beliefs of clinicians. For example, 90% (86%–94%) of respondents from each hospital reported that current practice on their unit was to obtain a BC in any PICU patient with a new fever. In comparison, only 26% (18%–31%) agreed that BCs should always be obtained for new fever.

Also, respondents indicated that the decision to order a BC was often made by fellow physicians [79% (75%–90%)] and attending physicians [66% (48%–80%)]. They tended to order BCs reflexively [83% (76%–91%)] without going to the bedside to examine patients [70% (62%–75%)]. Nurses were not often included in the decision-making process [34% (27%–41%)]. The main perceived barriers to reducing unnecessary BC use in PICU patients included fear of missing bacterial sepsis [83% (76%–90%)] and difficulty in standardizing clinical practices across different clinicians [61% (57%–70%)]. There were significant differences in the responses to the WSA survey across hospitals and clinicians, as previously described.^9^

### Results of WSA Survey Evaluation

**Fig. 1.**
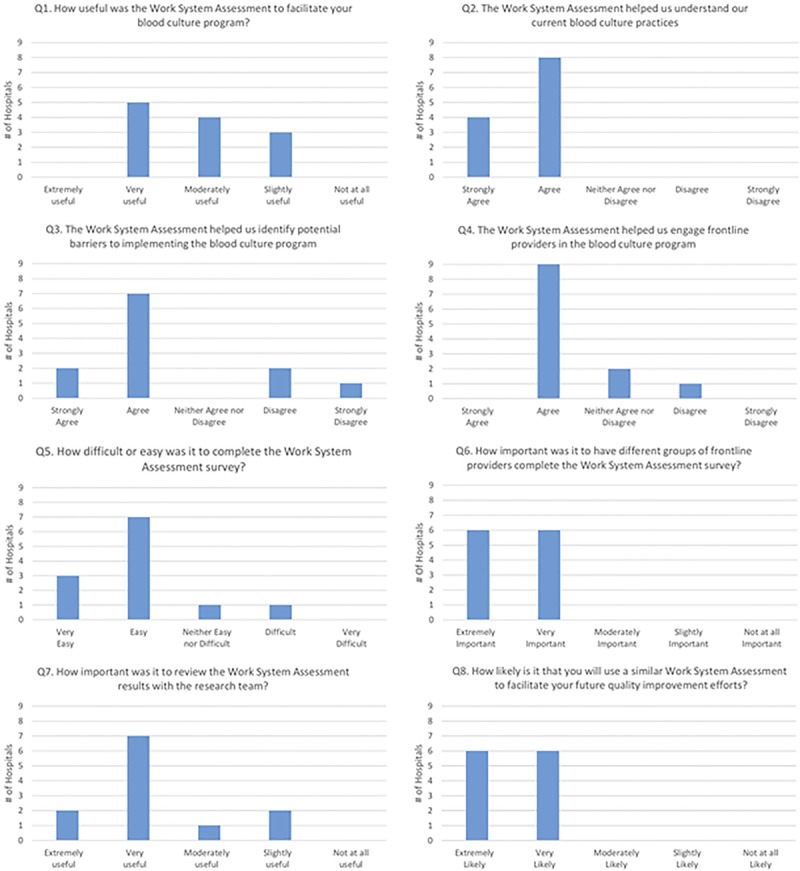
Evaluation of the WSA survey.

Physician champions at 12 of the 15 hospitals evaluated the use of the survey-based WSA. Figure 1 shows the evaluation results.

In general, the survey-based WSA was found useful in facilitating the dissemination of the program. All 12 hospitals (100%) strongly agreed or agreed that the survey-based WSA helped them understand their current BC ordering practices (“It did help me learn a lot about the unit that I did not already know.”). Also, 9 hospitals (75%) strongly agreed or agreed that the survey-based WSA facilitated the identification of potential barriers to implementing the program [“It helped to use (identified barriers) to propel forward the conversation.”] and the engagement of clinicians in the program (“People felt appreciative that their opinion was asked.”). Hospitals, however, indicated that the WSA survey was not comprehensive enough to address every aspect of BC use (“There was nothing about how technology is used to order BCs.”).

Ten hospitals (83%) considered the WSA survey very easy or easy to complete. The importance of having different clinicians complete the WSA survey (“It was important to get all the pieces of information from everyone.”) and reviewing the WSA results with the research team [“(The research team) helped us go through barriers and ways we could do to (address those barriers).”] was highlighted by all 12 hospitals (100%). Finally, all 12 hospitals (100%) indicated that they would use a similar WSA to facilitate future QI efforts [“(We) have not done (WSA) in the past, but would definitely do it in the future.”].

## DISCUSSION

Broad and rapid dissemination of successful QI interventions is critical but challenging. A toolkit with overarching dissemination principles, detailed steps of the dissemination process, and specific tools to be used in the dissemination process is needed for large-scale intervention dissemination.^5^ Although various frameworks have been proposed to describe the principles and processes of large-scale dissemination,^10–12^ limited practical tools are available to address the unique challenges of large-scale dissemination. This article highlights the importance and challenges of doing a WSA in large-scale dissemination and demonstrates how a survey-based tool could facilitate a WSA in the dissemination of a program to improve BC use to a multisite collaborative.

The dissemination of any QI program requires a clear understanding of the discrepancies between the tasks prescribed by the program and the tasks performed at individual settings.^13^ Both the WSA survey data and the evaluation of the WSA survey showed that the survey-based WSA could help detect such discrepancies. A few hospitals indicated that certain aspects of the BC ordering process were not included in the WSA survey, which could be an intrinsic disadvantage of any survey tool. Trade-offs regarding the content of the survey always have to be made. Based on our experience, the in-depth qualitative interviews were necessary to inform the design of the WSA survey.

Also, the survey-based WSA provided an efficient way to examine different perspectives. As shown by the WSA survey data, different clinicians had different opinions about the current and ideal BC ordering practices. The survey-based WSA ensured the consideration and integration of these different opinions and boosted the early engagement of different clinicians in the dissemination process. During the evaluation of the WSA survey, a few hospitals reflected the drawbacks of not surveying pediatric specialists who played a critical role in BC ordering. Future efforts using survey-based WSA need to identify all key stakeholders to be surveyed carefully. The survey-based WSA itself can also be a tool to help identify relevant stakeholders.

Finally, besides assessing the work systems and processes of individual hospitals, the survey-based WSA enabled cross-hospital comparison and learning. Comparing and sharing the WSA survey data across the collaborative helped create isomorphic pressures^14^ and peer-to-peer learning opportunities,^15^ both of which were critical to facilitating the dissemination of the program. The research team played an essential role in summarizing, sharing, reviewing, and discussing the WSA survey data with the participating hospitals. Although the involvement of researchers is not a necessary condition for the successful use of the survey-based WSA, someone has to take the responsibility to coordinate and manage the entire process, including the design and administration of the WSA survey, and the analysis and sharing of the survey-based WSA data.

## CONCLUSIONS

Assessment of the local environment is essential for the successful dissemination of QI interventions. The time and resources that WSA requires, however, pose significant challenges to its application to large-scale dissemination. We developed and pilot tested a survey-based tool to facilitate WSA in large-scale dissemination. The use of the survey-based tool was contingent not only on its design and administration but also on how the results were shared with and used by individual hospitals. Future research is needed to expand the application of the survey-based WSA tool and develop additional tools to address other challenges of large-scale dissemination.

## DISCLOSURE

Dr. Xie receives support from the Centers for Disease Control and Prevention (R01CE003150). Dr. Milstone receives support from the National Institute of Health (K24AI141580). Dr. Woods-Hill receives support from the National Institute of Health Ruth L. Kirschstein National Research Service Award (T32HL098054-55). The other authors have no financial interest to declare in relation to the content of this article.

## ACKNOWLEDGMENTS

We thank the quality improvement (QI) team and pediatric intensive care unit (PICU) clinicians at each hospital for their participation in the *B*lood Cultu*r*e *I*mprovement *G*uidelines and Diagnos*t*ic *St*ewardship for *A*ntibiotic *R*eduction in Critically Ill Children (Bright STAR) collaborative and their assistance with the work system assessment (WSA) survey.

## Supplementary Material


