# Supplementary material for: Survey-based Work System Assessment to Facilitate Large-scale Dissemination of Healthcare Quality Improvement Programs
Source: Pediatr Qual Saf. 2020 Apr 9;5(2):e288. doi: 10.1097/pq9.0000000000000288 (PMC7190253; doi:10.1097/pq9.0000000000000288)
Supplement: Supplementary file 1 [file pqs-5-e288-s001.docx]

Supplement 1. The electronic interview distributed to participants, annotated to indicate questions chosen for analysis by clinician type, experience, and sites’ baseline blood culture rate

Dear Bright STAR participants,
 
 We are working with clinicians from your hospital to improve blood culturing practices. This survey will take less than 10 minutes to complete and will inform a quality improvement program in your ICU. Completing this survey is strictly voluntary and your responses are confidential. Your completion of this survey will serve as your consent to be in this research study.  All results will be reported in aggregate form so that no one person can be identified. Your input is invaluable to the success of this project.
Thank you from the Bright STAR team. 
For questions, please email Dr. Aaron Milstone and Dr. Charlotte Woods-Hill at brightstar@jhmi.edu

**Annotation key:**

^a^ = responses to this item analyzed by sites’ baseline blood culture rate

^b^ = responses to this item analyzed by respondent type and experience

| 1. **^a b^Q1 In general, in your PICU, how likely is it that blood cultures are ordered in the following scenarios:** | Extremely likely (1) | Somewhat likely (2) | Neither likely nor unlikely (3) | Somewhat unlikely (4) | Extremely unlikely (5) |
| --- | --- | --- | --- | --- | --- |
| Fever, new (new fever=afebrile in preceding 48hrs) (1) |  |  |  |  |  |
| Fever, persistent (2) |  |  |  |  |  |
| Fever, patient with central venous catheter (3) |  |  |  |  |  |
| Hypothermia, new (4) |  |  |  |  |  |
| Hypotension, new (5) |  |  |  |  |  |
| New onset poor perfusion on physical exam (6) |  |  |  |  |  |
| Positive blood culture in preceding 24hrs (7) |  |  |  |  |  |
| Request by another clinical team (such as Oncology, Surgery, or Infectious Disease) (8) |  |  |  |  |  |
| Surveillance (e.g. asymptomatic, screening culture in high risk groups) (9) |  |  |  |  |  |
| Elevated WBC, new (10) |  |  |  |  |  |
| Elevated CRP, new (11) |  |  |  |  |  |

**^a b^Q2 What are your *preferred sources* for obtaining a blood culture on a patient with a *First/New* Fever (new fever=afebrile in preceding 48hrs)? (Assume patient has a central venous catheter, arterial catheter, and dialysis catheter in place).** 

Please check **ALL** that apply.
 (Leave all options unchecked if you would not order a blood culture in any of the below scenarios).
  

- Peripheral venipuncture (1)
- Central venous catheter lumen 1 (2)
- Central venous catheter lumen 2 (3)
- Central venous catheter lumen 3 (4)
- Arterial catheter (5)
- Dialysis Catheter (6)

**^a b^Q3 What are your preferred sources for obtaining a blood culture on a patient with a *persistent fever?* (Assume patient has a central venous catheter, arterial catheter, and dialysis catheter in place).** 
Please check ALL that apply.
(Leave all options unchecked if you would not order a blood culture in any of the below scenarios).

- Peripheral venipuncture (1)
- Central venous catheter lumen 1 (2)
- Central venous catheter lumen 2 (3)
- Central venous catheter lumen 3 (4)
- Arterial catheter (5)
- Dialysis Catheter (6)

| **^a b^Q4  To what extent do you agree or disagree with the following statements?** | Strongly agree (1) | Agree (2) | Neither agree nor disagree (3) | Disagree (4) | Strongly disagree (5) |
| --- | --- | --- | --- | --- | --- |
| Blood cultures can have unintended negative consequences (1) |  |  |  |  |  |
| All PICU patients with new fever should get a blood culture (2) |  |  |  |  |  |
| All PICU patients with new fever and a central venous catheter should get a blood culture (3) |  |  |  |  |  |
| Blood cultures are likely to be positive for bacteria when they are drawn from febrile patients (4) |  |  |  |  |  |
| Blood cultures are ordered too frequently in our unit (5) |  |  |  |  |  |
| The decision to order a blood culture is often made by **resident physicians** (6) |  |  |  |  |  |
| The decision to order a blood culture is often made by **nurse practitioners** (7) |  |  |  |  |  |
| The decision to order a blood culture is often made by **fellow physicians** (8) |  |  |  |  |  |
| The decision to order a blood culture is often made by **attending physicians** (9) |  |  |  |  |  |
| Nurses are included in the decision making process to order a blood culture (10) |  |  |  |  |  |
| Pediatric specialists (e.g. oncologists, infectious disease) play an important role re: blood culture decision making (11) |  |  |  |  |  |
| Clinicians always review vital signs and other clinical data (e.g. labs, x-rays) before ordering a blood culture (12) |  |  |  |  |  |
| Clinicians order blood cultures reflexively in response to signs and symptoms such as fever or hypotension (13) |  |  |  |  |  |
| Clinicians always perform a physical exam before ordering a blood culture (14) |  |  |  |  |  |
| Among different **clinicians**, blood culture ordering practices vary (i.e. decision about culture source, reason for getting a culture, frequency with which to repeat blood cultures) (15) |  |  |  |  |  |
| Among different **shifts**, blood culture ordering practices vary (i.e. decision about culture source, reason for getting a culture, frequency with which to repeat blood cultures) (16) |  |  |  |  |  |
| Clinicians change their blood culture practices when working with different patient populations (e.g. oncology patients, surgery patients, etc.) (17) |  |  |  |  |  |

| **^a b^Q5 How likely is it that the following items will act as potential barriers to reducing the number of blood cultures ordered in your clinical practice setting?** | Extremely likely (1) | Somewhat likely (2) | Neither likely nor unlikely (3) | Somewhat unlikely (4) | Extremely unlikely (5) |
| --- | --- | --- | --- | --- | --- |
| Opinion of consulting service or other clinicians (1) |  |  |  |  |  |
| The decision making process for ordering blood cultures is rarely discussed among physicians (2) |  |  |  |  |  |
| The decision making process for ordering blood cultures is rarely discussed between physicians and nurses (3) |  |  |  |  |  |
| Many clinicians have varied practices that will be difficult to standardize (4) |  |  |  |  |  |
| Some clinicians may resist efforts to standardize practice (5) |  |  |  |  |  |
| Lack of scientific evidence that blood culture ordering frequency can be safely reduced (6) |  |  |  |  |  |
| Concern for potentially missing sepsis (7) |  |  |  |  |  |
| It is difficult to obtain all pertinent information for blood culture decision making from the Electronic Medical Record (8) |  |  |  |  |  |
| The physical layout of our unit prevents clinicians from doing a physical exam before ordering a blood culture (9) |  |  |  |  |  |

Q6 Enter additional potential barriers to reducing the number of blood cultures ordered in your clinical practice setting, if any:

________________________________________________________________

**Q7 How likely is it that the following items will act as potential barriers to obtaining peripheral blood cultures in your clinical practice setting?**

|  | Extremely likely (1) | Somewhat likely (2) | Neither likely nor unlikely (3) | Somewhat unlikely (4) | Extremely unlikely (5) |
| --- | --- | --- | --- | --- | --- |
| Ability to get timely peripheral venipuncture completed (e.g. waiting for phlebotomy service or other trained professional) (1) |  |  |  |  |  |
| Pain/discomfort for child versus painless draw from existing catheter (2) |  |  |  |  |  |
| Opinion of consulting service or other clinicians (3) |  |  |  |  |  |
| Lack of evidence that peripheral blood cultures have added yield/utility over central line cultures alone (4) |  |  |  |  |  |
| Many clinicians have varied practices that will be difficult to standardize (5) |  |  |  |  |  |
| Some clinicians may resist efforts to standardize practice (6) |  |  |  |  |  |

**Q8 Enter additional potential barriers to obtaining peripheral blood cultures in your clinical practice setting, if any:**

________________________________________________________________

**Q9 How useful would the following intervention tools be in improving blood culture practices in your PICU?**

|  | Extremely useful (1) | Very useful (2) | Moderately useful (3) | Slightly useful (4) | Not at all useful (5) |
| --- | --- | --- | --- | --- | --- |
| A checklist of clinical information that clinicians need to review before ordering a blood culture (1) |  |  |  |  |  |
| A decision algorithm guiding the selection of the source(s) for obtaining a blood culture (2) |  |  |  |  |  |

**Q10 How would you prefer a checklist and/or algorithm be implemented in your PICU?  Check ALL that apply**

- Electronic Medical Record tool, if possible (1)
- Paper-based format posted in unit work areas (2)
- Presented to clinicians in didactic sessions (3)
- Integrated into clinician hand-off process (4)
- Other (Enter response) (5) ________________________________________________

**Q11 Bright STAR Site Name**

**Q12 Which of the following describes your current role?**

- Attending Physician (1)
- Fellow Physician (2)
- Resident Physician (3)
- Nurse Practitioner (4)
- Registered Nurse (5)
- Physician Assistant (6)
- Hospitalist (8)
- Other (please specify) (7) ________________________________________________

**Q13 How long have you been in your current role (years)**

________________________________________________________________

Thank you very much for participating in this survey!
If you have any questions or concerns, please send us an email: brightstar@jhmi.edu
